# Environmental Conditions Outweigh Geographical Contiguity in Determining the Similarity of *nifH*-Harboring Microbial Communities in Sediments of Two Disconnected Marginal Seas

**DOI:** 10.3389/fmicb.2016.01111

**Published:** 2016-07-20

**Authors:** Haixia Zhou, Hongyue Dang, Martin G. Klotz

**Affiliations:** ^1^State Key Laboratory of Marine Environmental Science, Institute of Marine Microbes and Ecospheres, and College of Ocean and Earth Sciences, Xiamen UniversityXiamen, China; ^2^Centre for Bioengineering and Biotechnology, College of Chemical Engineering, China University of Petroleum (East China)Qingdao, China; ^3^Department of Food Quality and Safety, College of Life Science, Dezhou UniversityDezhou, China; ^4^Department of Biology and School of Earth and Environmental Sciences, Queens College, City University of New YorkQueens, NY, USA

**Keywords:** coastal sediments, ecophysiological specialization, estuary, marginal sea, niche differentiation, *nifH*, nitrogen fixation, sulfate-reducing bacteria

## Abstract

Ecological evidence suggests that heterotrophic diazotrophs fueled by organic carbon respiration in sediments play an important role in marine nitrogen fixation. However, fundamental knowledge about the identities, abundance, diversity, biogeography, and controlling environmental factors of nitrogen-fixing communities in open ocean sediments is still elusive. Surprisingly, little is known also about nitrogen-fixing communities in sediments of the more research-accessible marginal seas. Here we report on an investigation of the environmental geochemistry and putative diazotrophic microbiota in the sediments of Bohai Sea, an eutrophic marginal sea of the western Pacific Ocean. Diverse and abundant *nifH* gene sequences were identified and sulfate-reducing bacteria (SRB) were found to be the dominant putative nitrogen-fixing microbes. Community statistical analyses suggested bottom water temperature, bottom water chlorophyll a content (or the covarying turbidity) and sediment porewater *Eh* (or the covarying pH) as the most significant environmental factors controlling the structure and spatial distribution of the putative diazotrophic communities, while sediment Hg content, sulfide content, and porewater ${\text{SiO}}_{3}^{2-}$-Si content were identified as the key environmental factors correlated positively with the *nifH* gene abundance in Bohai Sea sediments. Comparative analyses between the Bohai Sea and the northern South China Sea (nSCS) identified a significant composition difference of the putative diazotrophic communities in sediments between the shallow-water (estuarine and nearshore) and deep-water (offshore and deep-sea) environments, and sediment porewater dissolved oxygen content, water depth and *in situ* temperature as the key environmental factors tentatively controlling the species composition, community structure, and spatial distribution of the marginal sea sediment *nifH*-harboring microbiota. This confirms the ecophysiological specialization and niche differentiation between the shallow-water and deep-water sediment diazotrophic communities and suggests that the *in situ* physical and geochemical conditions play a more important role than geographical contiguity in determining the community similarity of the diazotrophic microbiota in marginal sea sediments.

## Introduction

N_2_-fixing prokaryotes play a key role in marine nitrogen (N) cycling and ecosystem functioning such as carbon sequestration by providing newly fixed nitrogenous nutrients, particularly important in oligotrophic environments of the ocean (Karl et al., 1997; Dore et al., 2002; Montoya et al., 2004; Steppe and Paerl, 2005; Karl and Letelier, 2008; Sohm et al., 2011). Marine N_2_ fixation studies have been conducted for more than half a century, yet many diazotrophic microbes, their ecophysiology and environmental response have been revealed only in recent decades (reviewed by Zehr and Kudela, 2011; Voss et al., 2013). The marine N cycle appears to be a conundrum because the estimated N input by N_2_ fixation is significantly lower than the estimated N loss *via* denitrification and anaerobic ammonium oxidation (anammox) (Mahaffey et al., 2005). This suggested that the inventory of reactive nitrogen in the oceans is unbalanced and dwindling (Moisander et al., 2014). However, this conundrum may be an artifact caused by uncertainties in previous research results and underestimations of N_2_-fixation contribution to the marine N budget. These include insufficient and inaccurate measurements of the N_2_ fixation rate (Deutsch et al., 2007; Großkopf et al., 2012), undiscovered N_2_-fixing microbes (Zehr et al., 2001, 2008; Pernthaler et al., 2008; Dekas et al., 2009; Zehr, 2011; Voss et al., 2013), overlooked robustness of N_2_-fixing physiology of environmental diazotrophs (Knapp, 2012), and overlooked and undiscovered N_2_-fixing environments such as nutrient-rich estuarine and coastal seas and marine sediments (Mehta et al., 2003; Pernthaler et al., 2008; Bonnet et al., 2013; Voss et al., 2013). Historically, marine N_2_ fixation was thought to be carried out mainly by aggregate-forming cyanobacteria such as *Trichodesmium* and important only in surface and near-surface waters of the ocean (Paerl, 1990; Dore et al., 2002; Karl et al., 2002; Bergman et al., 2013). However, the water column below the photic layer has been found to harbor significant N_2_ fixation activities in many marine waters, which were mainly carried out by heterotrophic diazotrophs either in hypoxic and anoxic environments or in marine particle-associated microenvironments (Farnelid et al., 2011, 2013; Fernandez et al., 2011; Jayakumar et al., 2012; Bird and Wyman, 2013; Rahav et al., 2013; Dang and Lovell, 2016). Furthermore, the recent detection of numerous diazotrophic bacteria and archaea in marine sediments led to the hypothesis that marine sediments might constitute an important environment for N_2_ fixation in the oceans (Pernthaler et al., 2008; Dang et al., 2009a, 2013a; Dekas et al., 2009, 2014, 2016; Fulweiler, 2009; Miyazaki et al., 2009).

Hypoxic and anoxic environments can be formed and maintains more easily in marine sediments than in the water column, particularly in eutrophic coastal seas. Nitrogen gas is abundant in marine sediments, partially provided by microbial denitrification and anammox processes (Dang et al., 2009b; Trimmer and Nicholls, 2009; Shao et al., 2014). Therefore, heterotrophic rather than cyanobacterial diazotrophy may play a dominant role of N_2_ fixation in marine sediments (Bertics et al., 2013; Dekaezemacker et al., 2013). N_2_ fixation by heterotrophic diazotrophs requires a high amount of cellular energy that is mainly provided by respiration of large amounts of organic carbon (Shanmugam et al., 1978; Brill, 1980; Dang and Jiao, 2014). It has been speculated that the low N_2_-fixing rates of heterotrophic diazotrophs in the open ocean is caused by the lack of sufficient metabolic energy due to the scarcity of bioavailable organic carbon (Moisander et al., 2014). On the other hand, estuarine and coastal sediments may support high rates of N_2_ fixation by heterotrophic diazotrophs due to enhanced phytoplankton labile organic matter production under the impact of anthropogenic eutrophication and terrigenous nutrient inputs (Herbert, 1999; Boesch, 2002; Smith, 2003). Moreover, iron, phosphorus or both have been found to be the key factors limiting microbial N_2_ fixation and export production in many waters of the oligotrophic open oceans (Sañudo-Wilhelmy et al., 2001; Mills et al., 2004; Moore et al., 2009, 2013; Boyd and Ellwood, 2010; Sohm et al., 2011; Jacq et al., 2014). In estuarine and coastal sediments, iron and phosphorus may be sufficiently abundant to support high rates of microbial N_2_ fixation (Street and Paytan, 2005; Homoky et al., 2013; Karl, 2014). Furthermore, it is evident that the sediment diazotrophic communities are highly resistant to the inhibition of high environmental ${\text{NH}}_{4}^{+}$ and ${\text{NO}}_{3}^{-}$ concentrations (McGlathery et al., 1998; Knapp, 2012; Bertics et al., 2013). It was thus reasonably hypothesized that the marginal sea sediments may prove to be key environments of N_2_ fixation. However, fundamental knowledge about the identities, diversity, biogeography, and controlling environmental factors of sediment N_2_-fixing microbes is still lacking.

**Figure 1:**
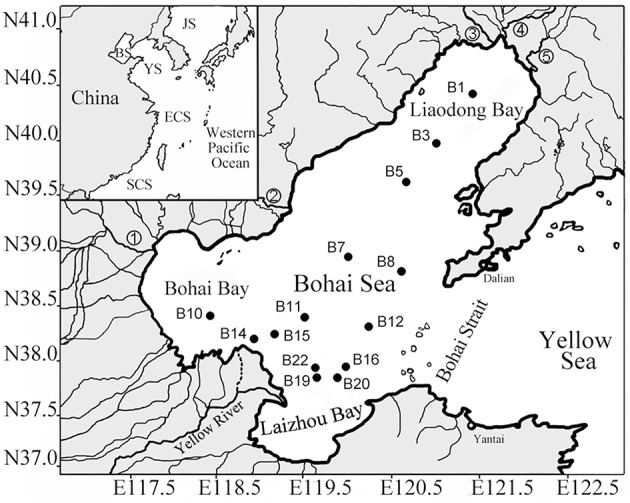
**Map of the Bohai Sea and sampling station sites**. The insert map shows the geographical location of the Bohai Sea in the western Pacific Ocean. Abbreviation and numerical symbols: BS, Bohai Sea; 1, Haihe River; 2, Luanhe River; 3, Dalinghe River; 4, Liaohe River; and 5, Daliaohe River (Dang et al., 2013b).

It has been reported that the sediments of the northern South China Sea (nSCS), a large and relatively oligotrophic marginal sea of the western Pacific Ocean, might harbor the highest diazotroph diversity among all the marine environments ever investigated using molecular ecology approaches targeting the *nifH* gene (encoding the nitrogenase reductase subunit) (Dang et al., 2013a). The Bohai Sea, another marginal sea of the western Pacific Ocean, is conversely characterized by its eutrophic status caused by high degree of river inputs and anthropogenic pollutions and by its low water exchange with the outer ocean due to its semi-enclosed topography (Figure 1; Dang et al., 2013b). To answer the question how diazotrophic communities vary and respond to distinct environmental conditions, sediment *nifH*-harboring microbial assemblages in the Bohai Sea were investigated in an environmental geochemistry context and comparatively analyzed against the putative diazotrophic assemblages of the nSCS.

## Materials and methods

### Site description, sample collection, and environment factor measurements

Bohai Sea is a large shallow water basin with an area of 77 × 10^3^ km^2^ and an average water depth of only 18 m. It is nearly enclosed and thus its water exchange capacity with the outer Yellow Sea of the western Pacific Ocean is highly limited (Figure 1). Moreover, more than 40 rivers discharge into the Bohai Sea. Thus, the Bohai Sea receives intense and extensive terrigenous and anthropogenic impacts, especially in its estuarine and coastal bay areas (PEMSEA and BSEMP, 2005).

Samples of surface sediments in the top 0–5 cm layer were collected from 14 stations of the Bohai Sea (Figure 1) in the August of 2008 as described previously (Dang et al., 2013b). At each station, seawater physicochemical and biological parameters (Supplementary Table S1) were measured *in situ* at various water depths with a Compact-CTD equipped with a TCDKU sensor (Alec Electronics, Japan) as reported in a previous publication (Dang et al., 2013b), which also contained most of the sediment and sediment porewater environmental parameters, except the sediment V and S contents that were measured in the current study via a VARIAN 725-ES inductively coupled plasma-optical emission spectrometer (Varian, Palo Alto, CA, USA) (Supplementary Table S1).

### DNA extraction and *nifH* gene clone library construction and analyses

Community genomic DNA of sediment microbes was extracted by using a FastPrep DNA Extraction Kit for Soil and a FastPrep-24 Cell Disrupter (MP Biomedicals, Solon, OH, USA) as previously described (Dang et al., 2013a,b). DNA concentrations were measured with dye PicoGreen (Molecular Probes, Eugene, OR, USA) and a Modulus Single Tube Multimode Reader fluorometer (Turner BioSystems, Sunnyvale, CA, USA). Partial *nifH* gene sequences were amplified with PCR primers nifHfw and nifHrv, which have a very broad coverage for both bacterial and archaeal *nifH* genes (Mehta et al., 2003). In order to test reproducibility of our experimental procedure and to identify any potential within-site variability of the sediment diazotrophic community, two separate *nifH* gene clone libraries (B22-1 and B22-2) were constructed for the sediments of sampling station B22, each from a distinct subcore DNA sample, respectively. PCR product cloning followed previous procedures (Dang et al., 2009a, 2013a). A miniprep method was used for recombinant plasmid extractions (Dang and Lovell, 2000). Cloned gene fragments were reamplified to check the correct size of the DNA inserts using cloning vector PCR primers M13-D and RV-M (Dang et al., 2008), which were also used for sequencing with an ABI 3770 sequencer (Applied BioSystems, Foster City, CA, USA). The obtained *nifH* gene sequences have been submitted to GenBank with accession numbers KM524369 to KM525656.

The *nifH* sequences were translated into conceptual protein sequences and the BLASTp program was used for retrieval of the top-hit NifH sequences from GenBank (last accessed 25 January 2014) (Altschul et al., 1997). The NifH protein sequences were grouped into operational taxonomic units (OTUs) using a 0.05 sequence distance cutoff calculated by using the DOTUR program (Schloss and Handelsman, 2005). These OTU sequences, along with reference sequences retrieved from GenBank, were aligned with program CLUSTAL X (version 2.1; Larkin et al., 2007), and used for phylogeny inference with the distance and neighbor-joining method implemented within the PHYLIP software package (version 3.69; Felsenstein, 1989), following a previously reported procedure (Dang et al., 2009a, 2013a).

### Quantification of sediment *nifH* gene copy numbers

The technique of real-time fluorescent quantitative PCR (qPCR) was employed to measure the abundance of the sediment *nifH* genes using primers nifHfw and nifHrv (Mehta et al., 2003; Dang et al., 2013a). Triplicate sediment DNA samples from each station were assayed with an ABI Prism 7500 Sequence Detection System (Applied Biosystems, Foster City, CA, USA), following a previously published SYBR Green qPCR protocol (Dang et al., 2013a). Plasmids containing the *nifH* gene inserts were extracted from *Escherichia coli* hosts using a Mini Plasmid Kit (Qiagen, Valencia, CA, USA), linearized with an endonuclease specific in the cloning vector region, and quantified using PicoGreen and a Modulus Single Tube Multimode Reader fluorometer (Dang et al., 2013a). A qPCR standard curve was then generated with serially diluted linearized plasmids containing the target *nifH* gene fragment.

The qPCR condition for *nifH* gene quantification was optimized based on a previous study (Dang et al., 2013a). The efficiency and sensitivity of the qPCR were shown in Supplementary Table S2, along with the range of the plasmid copy numbers used for the qPCR standard curve construction. In all experiments, negative controls lacking template DNA were run with the same qPCR procedure to prevent contamination or carryover. Agarose gel electrophoresis and melting curve analysis were performed to confirm the specificity of the qPCR reactions.

### Statistical analyses

Clone library coverage (*C*) was calculated as *C* = [1 − (*n*_1_/*N*)] × 100, where *n*_1_ is the number of unique OTUs and *N* the total number of clones in a library (Mullins et al., 1995). Shannon-Wiener (*H*), Simpson (*D*), and evenness (*J*) indices were calculated with the OTU data (Dang et al., 2013a). The DOTUR program (Schloss and Handelsman, 2005) was used for clone library rarefaction analysis and to calculate the abundance-based coverage estimator (*S*_ACE_) and the bias-corrected richness estimator Chao1 (*S*_Chao1_). The Fast UniFrac program (Hamady et al., 2010) was used for *nifH*-harboring microbial community clustering and principal coordinates analyses (PCoA). The Canoco software (version 4.5, Microcomputer Power, Ithaca, NY, USA) was used for the canonical correspondence analysis (CCA) (ter Braak and Šmilauer, 2002; Lepš and Šmilauer, 2003), to investigate any correlations between the *nifH*-harboring microbial communities and environmental factors by following a previously described procedure (Dang et al., 2013a). The SPSS software (version 17.0) was used for Pearson correlation analyses (significance level α = 0.05) of the sediment *nifH* gene abundance with environmental factors (Dang et al., 2013a).

The Fast UniFrac program (Hamady et al., 2010) was also used for clustering analyses of the sediment *nifH*-harboring microbial communities in both the Bohai Sea and the previously studied nSCS (Dang et al., 2013a), to identify larger geographical-scale characteristics of the putative diazotrophic microbiota. CCA analysis (ter Braak and Šmilauer, 2002; Lepš and Šmilauer, 2003) was also performed to tentatively identify the key environmental factors that may control the biogeographical distribution of the sediment *nifH*-harboring microbial communities in both marginal seas.

## Results

### Diversity of *nifH* gene sequences from bohai sea sediments

Community clustering (Supplementary Figure S1) and PCoA (Supplementary Figure S2) analyses using Fast UniFrac software revealed that the two *nifH* gene clone libraries (B22-1 and B22-2) constructed from separate sediment subcore samples of station B22 were highly similar. These statistical results demonstrated the reproducibility of our experimental procedures and the negligibility of within-site variability of the putative diazotrophic community detected by using the *nifH* gene biomarker. Therefore, in subsequent analyses, these two *nifH* gene clone libraries (B22-1 and B22-2) were pooled as a single B22 clone library.

Of all the 14 *nifH* gene clone libraries constructed, a total of 1309 clones were found to contain a valid *nifH* gene fragment, which were further identified as 1288 unique *nifH* DNA sequences yielding 619 deduced unique NifH protein sequences and 246 OTUs. Based on the obtained biodiversity index values (Table 1), the sediment *nifH* genes were highly diverse and their richness was heterogeneously distributed in the different sampling stations of the Bohai Sea. The high diversity (Supplementary Figure S3, Table 1) indicated that the sequences in the constructed *nifH* gene clone libraries might only represent the dominant putative diazotrophs in the Bohai Sea sediments. In general, the coastal site B15 had the lowest *nifH* gene diversity and the sites B5, B16, B20, and B22 might have the highest *nifH* gene diversity (Supplementary Figure S3, Table 1).

**Table 1:** **Biodiversity and predicted richness of the sediment ***nifH*** gene sequences obtained from the sampling stations of the Bohai Sea**.

**Station**	**No. of clones**	**No. of unique sequences**	**No. of OTUs**	***C* (%)**	***H***	**1/*D***	***J***	***S*_ACE_**	***S*_Chao1_**
B1	82	63	40	69.51	4.86	28.38	0.91	94.49	77.50
B3	80	58	44	63.75	5.05	31.60	0.92	96.33	102.00
B5	85	59	48	61.18	5.17	36.06	0.93	141.89	114.00
B7	87	58	35	73.56	4.39	15.72	0.86	101.42	77.17
B8	92	63	44	71.74	5.08	36.40	0.93	86.16	90.43
B10	88	58	41	71.59	4.82	23.92	0.90	96.60	83.86
B11	83	54	34	73.49	4.35	14.80	0.86	97.45	72.50
B12	89	61	37	74.16	4.51	15.92	0.87	75.28	79.17
B14	85	55	36	75.29	4.53	16.76	0.88	73.26	62.25
B15	91	57	27	84.62	3.89	10.06	0.82	54.73	45.20
B16	93	71	47	68.82	5.14	36.56	0.93	104.87	87.60
B19	86	63	37	73.26	4.60	19.86	0.88	91.17	73.14
B20	83	61	48	59.04	5.17	35.82	0.93	154.44	118.13
B22	185	111	66	78.38	5.22	24.38	0.86	143.74	152.67
B22-1	96	70	50	66.67	5.16	32.11	0.91	126.55	91.30
B22-2	89	65	40	70.79	4.73	21.52	0.89	98.8	105.00

### Phylogeny of deduced NifH protein sequences from bohai sea sediments

The obtained 1288 unique *nifH* gene sequences shared 37.5–99.7% sequence identity with one another and 51–99% sequence identity with the top-match sequences obtained from GenBank. Interestingly, the majority (87.7%) of the queried Bohai Sea sediment *nifH* gene sequences resulted in *nifH* top-match sequences derived from samples obtained from nSCS marine sediments (Dang et al., 2013a). The deduced 619 unique NifH protein sequences shared 33.0–99.2% sequence identity with one another and shared 53–100% sequence identity with the top-match GenBank NifH sequences. More than half (57.4%) of the 619 unique NifH sequences shared >90% sequence identity with known bacteria, such as *Alphaproteobacteria, Gammaproteobacteria, Deltaproteobacteria, Chlorobi, Firmicutes*, and *Verrucomicrobia* (Supplementary Table S3). Importantly, most of these bacteria with top-match NifH sequences are known diazotrophs. This result indicates that diverse obligately anaerobic sulfate-reducing bacteria (SRB) in the *Deltaproteobacteria* class, which accounted for 18.3% of the total OTUs and 48.2% of the total clones, probably constituted the majority of taxa of diazotrophic communities in Bohai Sea sediments (Supplementary Table S3, Supplementary Figure S4).

**Figure 2:**
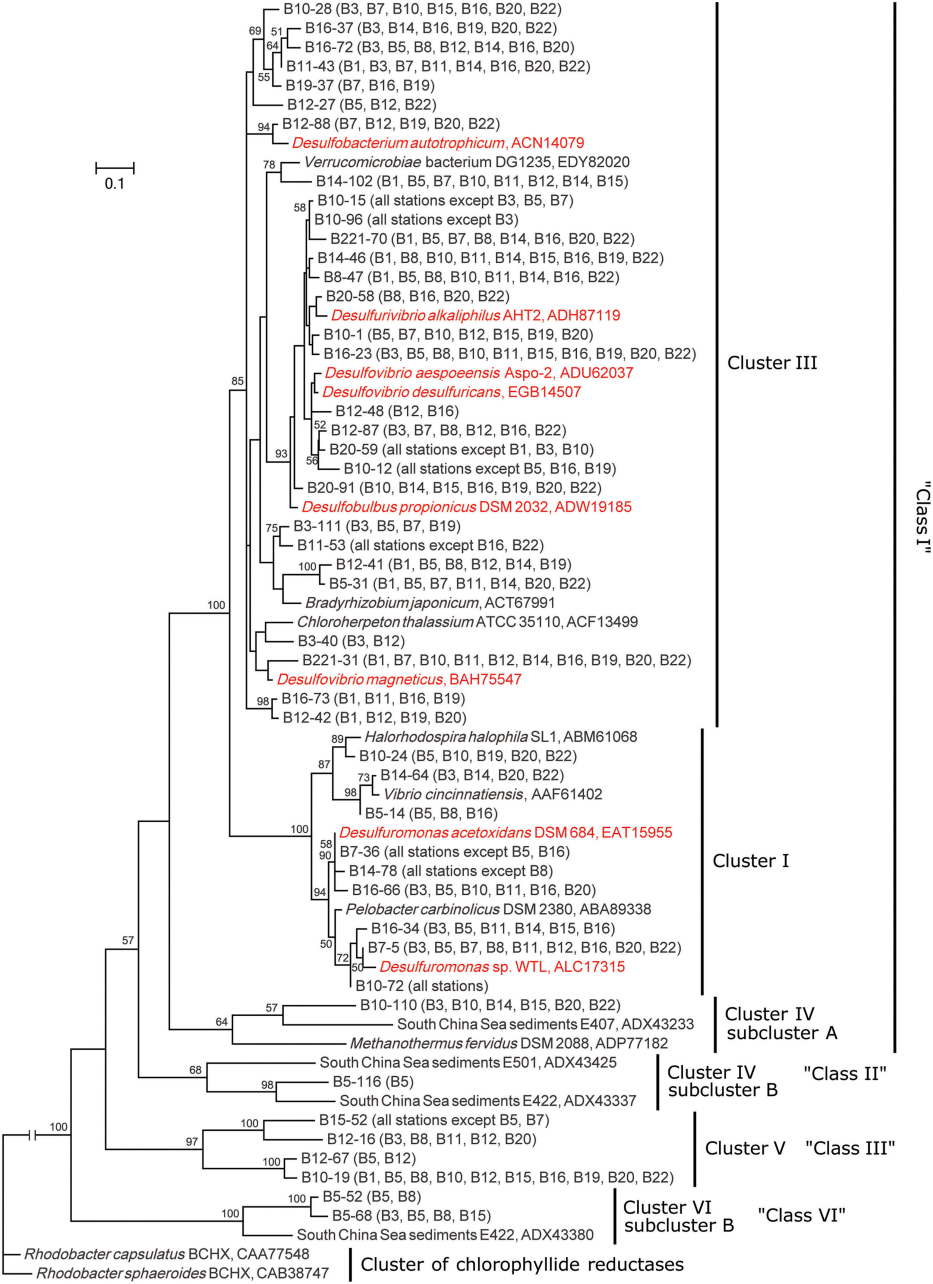
**Phylogenetic tree of the major Bohai Sea sediment NifH sequences constructed with the neighbor-joining method**. The NifH sequences of the most abundant OTUs (no <5 clones) were used for tree construction. The tree branch distances represent the amino acid substitution rate, and the scale bar represents the expected number of changes per homologous position. Bootstrap values higher than 50% of 100 resamplings are shown near the corresponding nodes. The chlorophyllide reductase iron protein subunit BchX sequences from *Rhodobacter capsulantus* and *R. sphaeroides* were used as outgroup.

The deduced NifH sequences were affiliated with six major groups in the constructed NifH phylogenetic tree (Figure 2, Supplementary Figure S4, Table 2), according to a recently proposed NifH sequence phylogenetic classification (Dang et al., 2009a, 2013a). Class I contained 141 OTUs and could be further divided into four clusters (Clusters I, II, III, and Cluster IV subcluster A), in which Cluster III was the largest containing 102 OTUs (Supplementary Figure S4). Clusters I and III were the only clusters that contained the NifH sequences obtained from all 14 sampling stations; therefore, these sequences tentatively represent the most prevalent diazotrophic microbes in the Bohai Sea sediments. Sequence Classes II, III, V, VI, and VIII represented 19, 32, 10, 43, and 1 OTUs (Supplementary Figure S4), respectively, and the pertinent putative diazotrophic microbes might thus be specifically distributed only in certain sediment environments of the Bohai Sea.

**Table 2:** **The detail composition of the sequences (and OTUs) in the NifH phylogenetic tree obtained from the Bohai Sea sediments**.

**Station**	**Class I**				**Class II**	**Class III**	**Class V**	**Class VI**	**Class VIII**
	**Cluster I**	**Cluster II**	**Cluster III**	**Cluster IV(A)**	**Cluster IV(B)**	**Cluster V**	**Cluster VI(A)**	**Cluster VI(B)**	
B1	28 (7)		41 (22)	1 (1)	2 (2)	6 (4)	2 (2)	2 (2)	
B3	24 (7)		37 (19)	6 (6)	4 (4)	6 (5)	1 (1)	2 (2)	
B5	23 (10)		33 (22)	3 (1)	11 (5)	2 (2)	3 (2)	10 (6)	
B7	38 (9)		41 (19)		4 (4)			4 (3)	
B8	17 (5)	1 (1)	37 (18)	2 (1)	4 (2)	21 (10)	3 (1)	7 (6)	
B10	26 (5)		45 (23)	4 (2)		5 (3)	1 (1)	6 (6)	1 (1)
B11	30 (6)		40 (16)		2 (2)	6 (6)		5 (4)	
B12	21 (4)		49 (22)		1 (1)	14 (8)	3 (1)	1 (1)	
B14	29 (7)		47 (22)	1 (1)		4 (2)		4 (4)	
B15	14 (5)		43 (14)	4 (2)	2 (2)	25 (2)		3 (2)	
B16	21 (7)		59 (28)		1 (1)	3 (3)	1 (1)	8 (7)	
B19	19 (8)		57 (21)	2 (2)	1 (1)	6 (4)	1 (1)		
B20	28 (10)		39 (25)	1 (1)	3 (2)	9 (7)		3 (3)	
B22	35 (10)		128 (44)	2 (2)	1 (1)	16 (6)	1 (1)	2 (2)	

### Key factors controlling the *nifH*-harboring microbial communities

**Figure 3:**
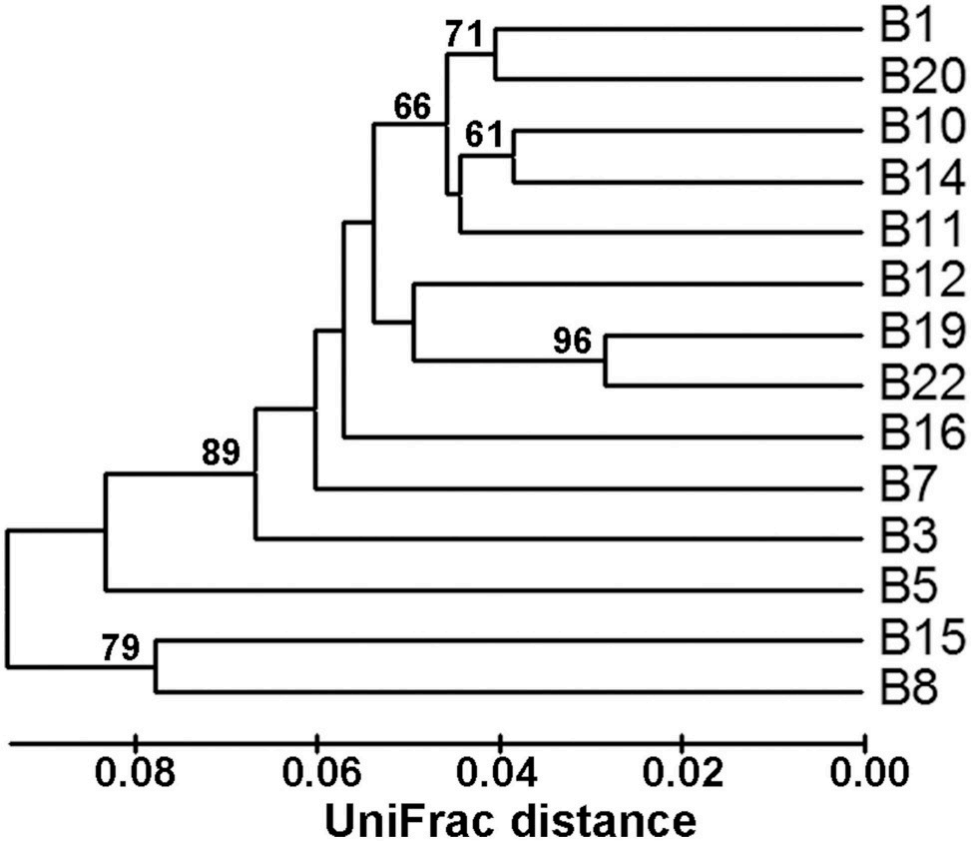
**Hierarchical clustering dendrogram of the Bohai Sea sediment ***nifH***-harboring microbial assemblages**. This dendrogram was constructed by using the Fast UniFrac weighted and normalized Jackknife Environment Clusters statistical method with the use of the NifH protein sequence data. The percentage supports of the classification tested with sequence jackknifing resamplings are shown near the corresponding nodes.

**Figure 4:**
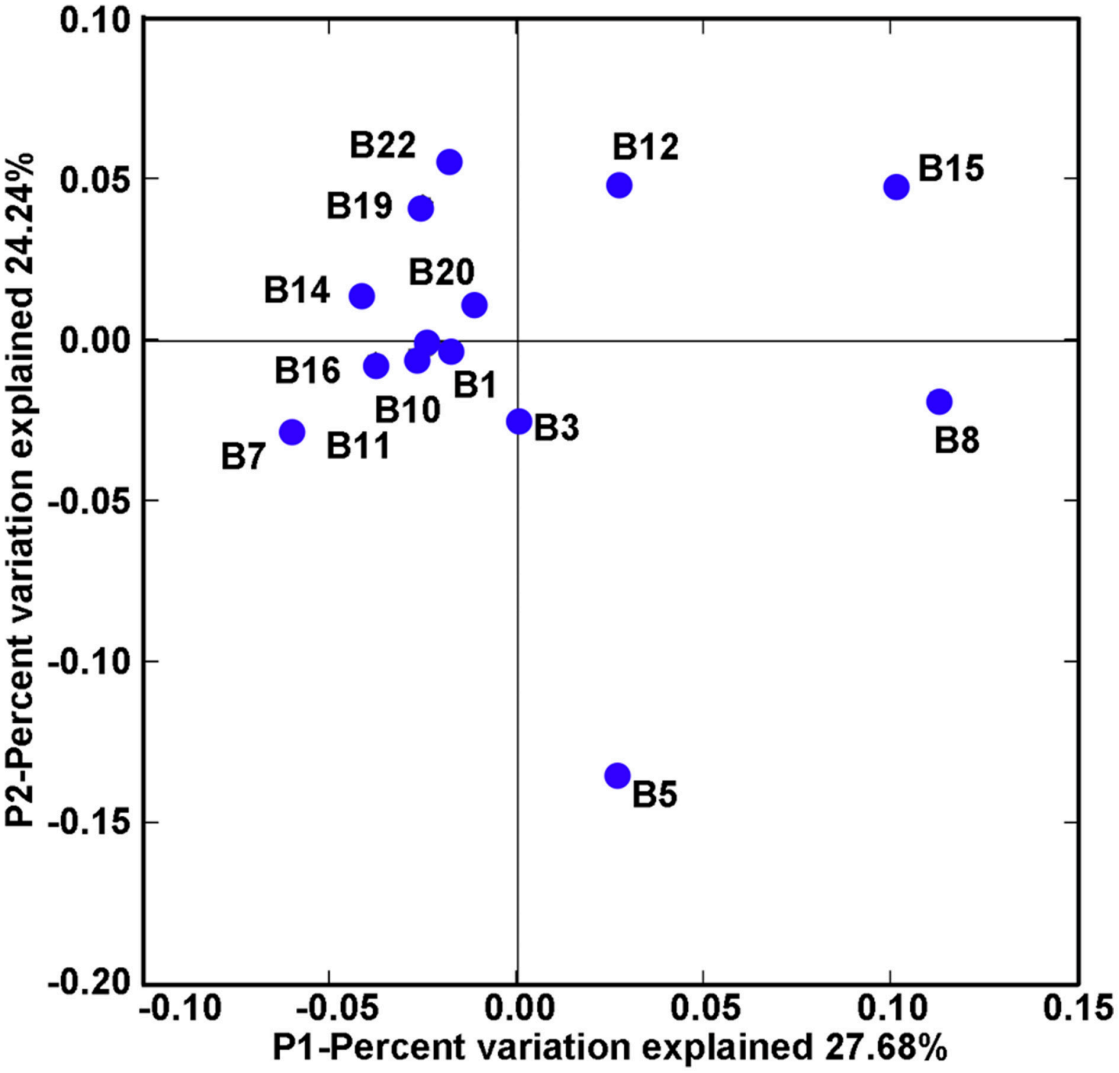
**PCoA ordination diagram of the Bohai Sea sediment ***nifH***-harboring microbial assemblages**. This diagram was produced by using the Fast UniFrac weighted and normalized PCoA method with the use of the NifH protein sequence data. The P1 and P2 show the percent variations of the diazotroph assemblages explained by the first two principal coordinates.

Community classification based on Fast UniFrac clustering analysis identified three distinct clusters of the *nifH*-harboring microbial assemblages in Bohai Sea sediments (Figure 3). The *nifH*-harboring microbial assemblages of stations B8 and B15 were grouped together and represented a distinct cluster, the assemblage of station B5 represented another distinct cluster formed by a singleton member, and the assemblages of the remaining stations were grouped together and formed a third distinct cluster. This community classification pattern of *nifH*-harboring microbial assemblages in the Bohai Sea sediments was further supported by the PCoA analysis (Figure 4).

CCA analysis was performed to decode the putative diazotroph-environment relationship in the Bohai Sea sediments (Supplementary Figure S5). Bottom water temperature (*p* = 0.001; 1000 Monte Carlo permutations), bottom water chlorophyll a content (or the covarying bottom water turbidity) (*p* = 0.015; 1000 Monte Carlo permutations) and sediment porewater *Eh* (or the covarying porewater pH; *p* = 0.032; 1000 Monte Carlo permutations) were identified as the most significant environmental factors that might control the structure and spatial distribution of the *nifH*-harboring microbial communities in Bohai Sea sediments.

### Abundance of the *nifH*-harboring microbes in bohai sea sediments

The qPCR results showed that the abundance of the *nifH* genes ranged from 2.95 × 10^7^ copies g^−1^ sediment (station B3) to 2.63 × 10^9^ copies g^−1^ sediment (station B19) in the Bohai Sea (Table 3). The highest *nifH* gene abundance occurred at the sampling station B1, B19, and B14, respectively, in Liaodong Bay, Laizhou Bay, and Bohai Bay sediments, respectively. Our previous study showed that the total bacterial abundance was also heterogeneously distributed in the sediments of the Bohai Sea, with the bacterial 16S rRNA genes ranging from 3.25 × 10^9^ copies g^−1^ sediment (station B16) to 2.10 × 10^10^ copies g^−1^ sediment (station B20) (Dang et al., 2013b). The ratios of the *nifH* gene abundance to the bacterial 16S rRNA gene abundance ranged from 0.36% (station B1) to 14.95% (station B19).

**Table 3:** **The abundance of the ***nifH*** and bacterial 16S rRNA genes in the sediments of the Bohai Sea**.

**Sampling station**	**Mean copy no. of target genes g**^−1^ **sediment (SE)**	
	***nifH***	Bacterial 16S rRNA^*^
B1	7.21 × 10^7^ (2.70 × 10^6^)	1.99 × 10^10^ (1.30 × 10^9^)
B3	2.95 × 10^7^ (2.65 × 10^6^)	5.04 × 10^9^ (2.09 × 10^8^)
B5	5.99 × 10^7^ (5.75 × 10^6^)	5.17 × 10^9^ (1.71 × 10^8^)
B7	1.45 × 10^8^ (3.07 × 10^6^)	8.00 × 10^9^ (6.19 × 10^8^)
B8	1.22 × 10^8^ (9.57 × 10^6^)	1.86 × 10^10^ (1.60 × 10^9^)
B10	2.77 × 10^8^ (1.01 × 10^7^)	1.45 × 10^10^ (1.33 × 10^9^)
B11	7.42 × 10^8^ (4.26 × 10^7^)	1.66 × 10^10^ (5.43 × 10^8^)
B12	8.70 × 10^8^ (7.46 × 10^7^)	1.33 × 10^10^ (2.82 × 10^8^)
B14	9.28 × 10^8^ (5.40 × 10^7^)	1.16 × 10^10^ (1.16 × 10^9^)
B15	4.34 × 10^8^ (3.88 × 10^7^)	1.06 × 10^10^ (8.64 × 10^8^)
B16	2.42 × 10^8^ (1.90 × 10^7^)	3.25 × 10^9^ (4.08 × 10^8^)
B19	2.63 × 10^9^ (2.22 × 10^8^)	1.76 × 10^10^ (7.52 × 10^8^)
B20	9.45 × 10^8^ (3.87 × 10^7^)	2.10 × 10^10^ (6.29 × 10^8^)
B22	5.54 × 10^8^ (4.14 × 10^7^)	1.83 × 10^10^ (6.02 × 10^8^)

* *The bacterial 16S rRNA gene abundance data were obtained from a previous study (Dang et al., 2013b)*.

Pearson correlation analyses indicated that the sediment Hg content (*p* = 0.018), sulfide content (*p* = 0.046), and porewater ${\text{SiO}}_{3}^{2-}$-Si content (*p* = 0.026) were positively correlated with the *nifH* gene abundance in all investigated Bohai Sea sediments.

### Comparison of *nifH*-harboring microbiota in bohai sea and nSCS sediments

**Figure 5:**
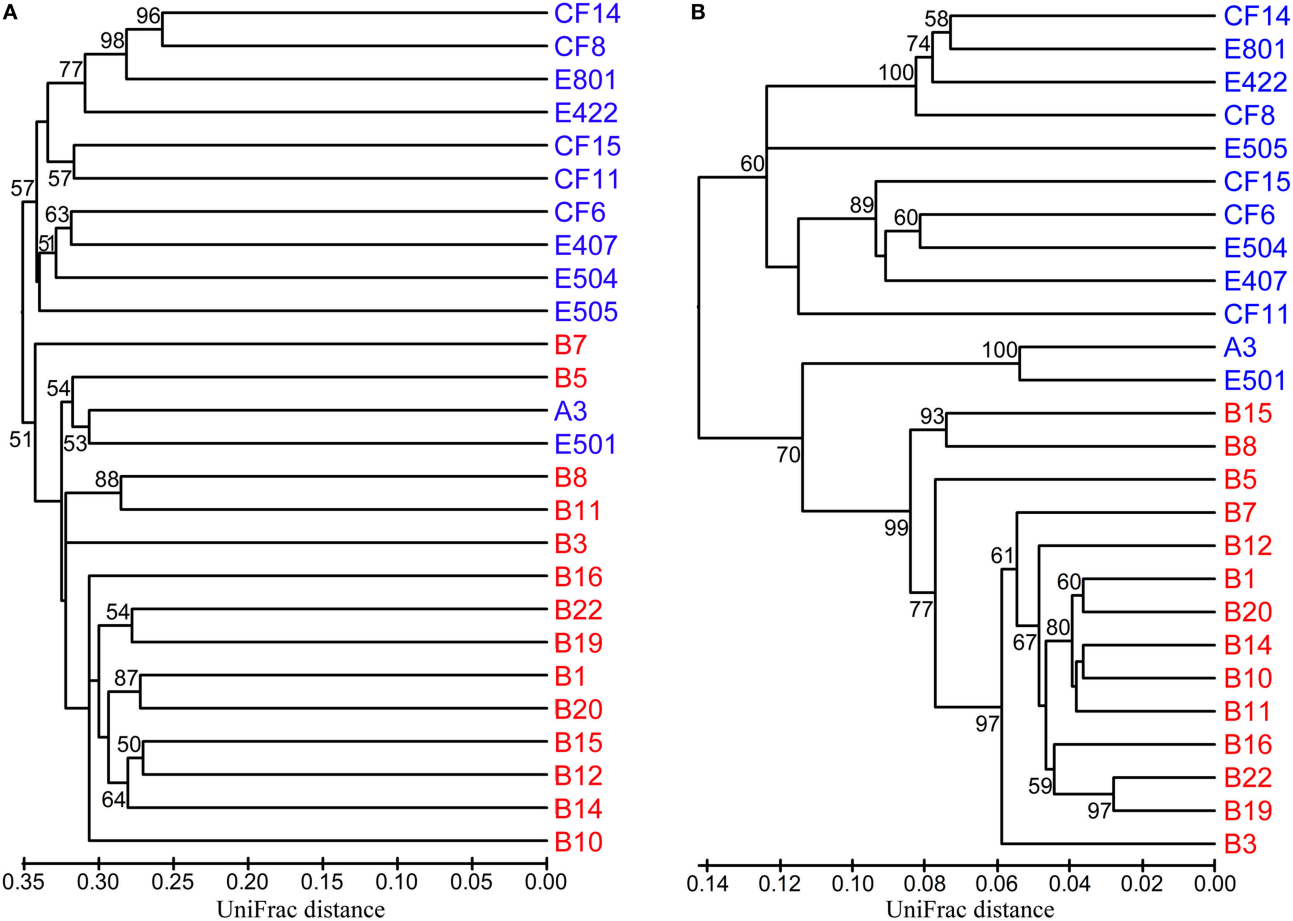
**Hierarchical clustering dendrograms of the Bohai Sea and northern South China Sea sediment ***nifH***-harboring microbial assemblages**. These dendrograms were constructed by using the Fast UniFrac unweighted **(A)** and weighted and normalized **(B)** Jackknife Environment Clusters statistical methods with the use of the NifH protein sequence data obtained from the current study and a previous study of the nSCS (Dang et al., 2013a). The percentage supports of the classifications tested with sequence jackknifing resamplings are shown near the corresponding nodes. The Bohai Sea sampling stations are labeled in red color and the northern South China Sea sampling stations are labeled in blue color.

**Figure 6:**
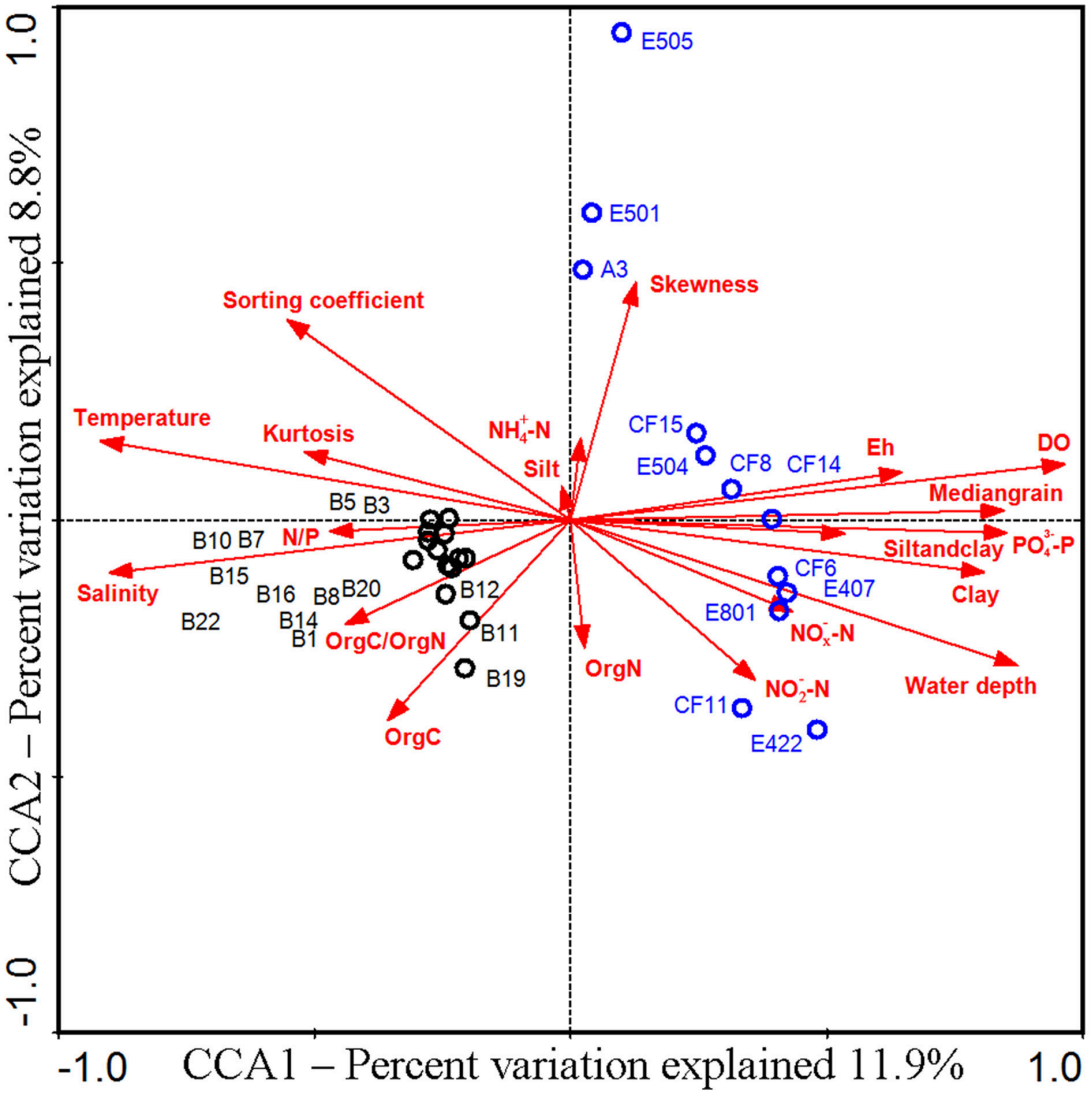
**CCA ordination diagram of the relationship between the sediment ***nifH***-harboring assemblages and environmental factors in the Bohai Sea and northern South China Sea**. This diagram was obtained by using the NifH OTU data obtained from the current study and a previous study of the northern South China Sea (Dang et al., 2013a).

In order to identify the difference of the diazotrophic microbial communities in sediments from distinct marine environments and to detect any general ecological characteristics of the diazotrophic microbiota in sediments of different marginal seas of the western Pacific Ocean, comparative analyses were made between sediment samples from the Bohai Sea and the previously studied nSCS (Dang et al., 2013a). Community classification analyses indicated that the *nifH*-harboring microbial communities could be classified into two groups based on both unweighted (Figure 5A) and weighted and normalized (Figure 5B) Fast UniFrac clustering analyses, with Group I containing exclusively the *nifH*-harboring microbial assemblages at the nSCS offshore and deep-sea sampling stations (water depth > 130 m) and Group II containing the *nifH*-harboring microbial assemblages at all the Bohai Sea sampling stations as well as the shallow-water sampling stations A3 and E501 of the nSCS (water depth < 70 m). The estuarine and nearshore sediments probably harbor N_2_-fixing microbial assemblages distinctly different from those of the offshore and deep-sea sediments, regardless of the large geographical distance between the Bohai Sea and the nSCS. The CCA analysis further identified the sediment porewater dissolved oxygen content (DO), water depth and *in situ* sediment temperature (the bottom water temperature was used to approximate the sediment temperature of the Bohai Sea) as the key environmental factors (*p* = 0.001, *p* = 0.002, and *p* = 0.014, respectively; 1000 Monte Carlo permutations) influencing significantly the community structure and biogeographical distribution of the putative sediment diazotrophic microbiota in Bohai Sea and nSCS sediments (Figure 6).

## Discussion

Nitrogenous nutrient-rich environments such as estuarine and coastal seawaters and marine sediments have long been regarded as environments lacking significant diazotrophic activities, inferred previously from bacterial culture-based physiological studies. This inferred popular opinion resulted in a long-time negligence of the N_2_-fixing microorganisms and activities in these environments whereas newer work led to the hypothesis that marginal sea sediments may instead harbor diverse and abundant N_2_-fixing microorganisms (Knapp, 2012; Bertics et al., 2013; Dang et al., 2013a; Dekaezemacker et al., 2013; Voss et al., 2013). To test this hypothesis, we probed the genetic potential of N_2_ fixation in marine sediment microbial communities: Indeed, highly diverse and abundant *nifH* gene sequences were obtained from the sediments of the Bohai Sea (Supplementary Figure S4, Table 2), consistent with the results obtained recently from marine sediments of the nSCS (Dang et al., 2013a). Many of the Bohai Sea *nifH* gene sequences are related to known bacterial diazotrophs (Supplementary Figure S4, Supplementary Table S3). While these results revealed the N_2_-fixation potential of microbial communities in Bohai Sea sediments, they also suggested microbial diazotrophy as a common ecological property of marginal sea sediments.

Besides being majorly related to environmental *nifH* gene sequences obtained mainly from the nSCS sediments (Dang et al., 2013a) and occasionally from salt marsh sediments (Lovell et al., 2001; Lovell and Davis, 2012), many of the *nifH* gene sequences from Bohai Sea sediments are related to diverse known bacterial species and a small number of methanogenic archaea (Supplementary Figure S4). This is consistent with previous findings that diazotrophic bacteria may be the predominant N_2_-fixing microbes in shallow water sediments (Burns et al., 2002). A recently defined cluster of archaeal *nifH* gene sequences, called Cluster III-x or Methane Seep Group, was associated with microbes frequently found in deep-sea methane seep, gas hydrate, and mud volcano sediments of the Pacific Ocean marginal seas (Pernthaler et al., 2008; Dang et al., 2009a, 2013a; Dekas et al., 2009, 2014, 2016; Miyazaki et al., 2009). However, Cluster III-x *nifH* gene sequences were not detected in samples obtained from Bohai Sea sediments. Because Cluster III-x *nifH* gene sequences are affiliated with anaerobic methane-oxidizing archaea (ANME), they may occur only in methane-rich environments. None of our Bohai Sea sampling stations were located in methane seeps, gas hydrates or mud volcanoes. The lack of the chemoautotrophic ANME *nifH* gene sequences in samples from shallow coastal sediments is consistent with the *in situ* geochemical condition of the Bohai Sea.

More than half of the deduced NifH sequences associated with Bohai Sea sediment samples shared high (>90%) identity with NifH sequences from known bacteria, of which the majority are SRB. This result indicates that SRB may be the dominant and prevalent N_2_-fixing microbes in the sediments of the Bohai Sea. Many SRB harbor functional N_2_ fixation genetic inventories (Barton and Fauque, 2009) and SRB were previously found to be important N_2_-fixing bacteria in marine intertidal microbial mats (Zehr et al., 1995; Steppe and Paerl, 2002, 2005; Stal et al., 2010). It was recently reported that the benthic microbial N_2_ fixation rate is coupled to sulfate reduction activities in lagoons and coastal bays supporting the notion that SRB may play a key role in sediment N_2_ fixation and new nitrogen production (Bertics et al., 2010, 2013). Furthermore, N_2_ fixation activities of sediment SRB are tolerant of high (e.g., up to 0.8 or 1.2 mM) environmental ${\text{NH}}_{4}^{+}$ concentrations (McGlathery et al., 1998; Bertics et al., 2013) suggesting that benthic N_2_ fixation is robust and widespread in marine sediments. In our current study, sediment sulfide, the product of microbial sulfate reduction, was found to correlate positively with *nifH* gene abundance in Bohai Sea sediments. This suggests that SRB may contribute substantially to N_2_ fixation and sulfate reduction in sediments and thus determine the abundance, spatial distribution, structure, and activity of diazotroph communities in marginal sea sediments.

SRB have been found to be the principal contributors to the accumulation and persistence of environmental organic Hg in coastal marine sediments (Sunderland et al., 2004; Schaefer et al., 2011; Parks et al., 2013). It is also known that sulfide produced by SRB facilitates precipitation and thus accumulation and persistence of Hg in anoxic marine sediments (Baeyens et al., 1998). These ecophysiological properties of SRB may explain the correlation between *nifH* gene abundance and Hg content in the studied Bohai Sea sediments. Hg is one of the key environmental contaminants in many estuaries and coastal bays of the Bohai Sea (Wang et al., 2010; Gao et al., 2014), where eutrophication is common, especially in summer times (Wang et al., 2009). Our data suggest that the accumulation and persistence of Hg in the sediments of the Bohai Sea may be attributable, at least partially, to the activity of *nifH*-harboring SRB.

The *in situ* bottom water temperature was putatively identified as a key environmental factor controlling the community structure and spatial distribution of the sediment putative diazotrophs in the Bohai Sea (Supplementary Figure S5). This finding agrees with the previously reported relationship of sediment *in situ* temperature with the community structure and spatial distribution of *nifH*-harboring microbial assemblages in the nSCS sediments (Dang et al., 2013a). Our current study supports the importance of temperature as a key environmental factor controlling universally the distribution of the diazotroph communities in marine water columns and sediments; also because temperature has previously been implicated as the most important environmental factor in the spatial distribution of seawater N_2_-fixing cyanobacteria in the oceans (Stal, 2009). Temperature influences not only nitrogenase activity but also O_2_ solubility in seawater and sediment porewater. Enhanced respiration is usually employed by diazotrophs under elevated O_2_ concentrations (such as under low temperature conditions) for providing energy to replace O_2_-damaged nitrogenase by *de novo* synthesis and/or for O_2_ consumption to maintain anaerobic N_2_-fixing activity (Stal, 2009; Großkopf and Laroche, 2012; Bandyopadhyay et al., 2013; Brauer et al., 2013). These biochemical and physiological mechanisms explain well the importance of environmental temperature on the community structure, distribution, and activity of marine diazotrophic microbiota.

The bottom water chlorophyll a content (or the covarying bottom water turbidity) was putatively identified as another key environmental factor controlling the community structure and spatial distribution of the putative diazotrophs in Bohai Sea sediments (Supplementary Figure S5). Chlorophyll a is related to the biomass and primary production of phytoplankton and both chlorophyll a and turbidity may indicate the potential of organic matter export from water column to marine sediments (Sobczak et al., 2002; Volkman and Tanoue, 2002). The positive correlation between *nifH* gene abundance with ${\text{SiO}}_{3}^{2-}$-Si content in Bohai Sea sediments suggests that diatom productivity affects size and activity of the diazotroph community in sediments (Yool and Tyrrell, 2003; Wei et al., 2004). The phylogenetic analysis of deduced NifH sequences (Supplementary Figure S4) suggests that most of the *nifH*-harboring microbes in Bohai Sea sediments are heterotrophic bacteria. Respiration of bioavailable organic carbon is the major process for heterotrophic diazotrophs to acquire the metabolic energy to fix N_2_ (Riemann et al., 2010; Moisander et al., 2014), which is highly energy demanding (Shanmugam et al., 1978; Brill, 1980). Therefore, organic carbon supplies, especially those from metabolizable phytoplankton and benthic diatom products, may constitute the major energy sources to fuel the obligately or facultatively anaerobic diazotrophic microbiota in sediments of coastal seas. In addition, obligately anaerobic bacteria such as SRB and, in particular, the process of N_2_ fixation, are highly sensitive to oxygen stress (Dixon and Kahn, 2004; Riemann et al., 2010; Zhou et al., 2011). Benthic respiration on organic matter consumes oxygen, which may thus help maintaining the hypoxic to anoxic state of the sediment environment and facilitate N_2_ fixation by anaerobic diazotrophs. In line with this, sediment porewater *Eh*, an indicator of the environmental redox status, was found to correlate significantly with the community structure and biogeographical distribution of *nifH*-harboring microbes in Bohai Sea sediments (Supplementary Figure S5). To maintain the hypoxic to anoxic condition may also be necessary for N_2_ supply in marine sediments: for instance, the heterotrophic N_2_ fixation process was linked directly to the N_2_ production processes by anammox and denitrifying bacteria in marine OMZ waters off the Peru coast (Loescher et al., 2014). A similar coupling of N_2_ fixation and N_2_ production microbial processes may also exist in marine sediments. Indeed, the Bohai Sea sediments harbor diverse anammox bacteria including a new species, *Candidatus* Scalindua pacifica (Dang et al., 2013b). Therefore, the *in situ* physical and geochemical condition, rather than the geographical location (Figures 3, 4, Supplementary Figure S5), may play a key role in determining the abundance, community structure, niche availability, biogeographical distribution, and activity of the diazotrophic microbiota in the sediment environments of the Bohai Sea.

In order to examine the general biogeographical and ecological characteristics of diazotrophic communities in sediments of the western Pacific Ocean, we performed a comparative analysis of *nifH*-harboring microbial communities in Bohai Sea and nSCS sediments (Dang et al., 2013a). The results of community classification analyses indicate that the estuarine and nearshore benthic environments harbor distinctly different putative diazotrophic assemblages from those of the offshore and deep-sea benthic environments (Figure 5). Furthermore, the consistency of the unweighted (only taking into account the NifH OTU composition for the analysis; Figure 5A) and weighted (taking into account both the composition of the NifH OTUs and their relative abundances for the analysis; Figure 5B) community classification results suggests that the difference in composition (Figure 5A) may be the key contributor to the difference between sediment diazotroph communities in the western Pacific Ocean. Although the A3 and E501 sampling stations in the nSCS are very distant and segregated from the Bohai Sea, the sediment diazotrophic assemblages of these two stations are separated from the remaining nSCS stations and clustered with the sampling stations of the Bohai Sea (Figure 5), indicating that the *in situ* physical and geochemical condition may play a more important role than geographical contiguity in determining the community similarity of diazotrophic microbiota in sediments. In line with this, water depth, sediment porewater DO and *in situ* temperature were identified as the key environmental factors tentatively controlling the composition, community structure and biogeographical distribution of the N_2_-fixing microbiota in the western Pacific Ocean sediments (Figure 6). The identification of sediment porewater DO as a controlling environmental factor further verifies the importance of the anaerobic heterotrophs in N_2_ fixation in marginal sea sediments, while the importance of water depth and *in situ* temperature suggests niche specialization and segregation between the shallow-water (estuarine and nearshore) and the deep-water (offshore and deep-sea) diazotrophic sediment communities. Our current investigation tentatively identified important general ecological, biogeochemical, and biogeographical characteristics and key environmental factors that influence the sediment diazotrophic microbiota and their potential activity in marginal seas.

## Author contributions

HD conceived and designed the experiments; HZ performed the experiments and analyzed the data; HD, HZ, and MK wrote the paper.

### Conflict of interest statement

The authors declare that the research was conducted in the absence of any commercial or financial relationships that could be construed as a potential conflict of interest.
