# Supplementary material for: Environmental Conditions Outweigh Geographical Contiguity in Determining the Similarity of nifH-Harboring Microbial Communities in Sediments of Two Disconnected Marginal Seas
Source: Front Microbiol. 2016 Jul 20;7:1111. doi: 10.3389/fmicb.2016.01111 (PMC4951488; doi:10.3389/fmicb.2016.01111)
Supplement: Supplementary file 1 [file DataSheet1.docx]

Supplementary Material

Environmental Conditions Outweigh Geographical Contiguity in Determining the Similarity of *nifH*-harboring Microbial Communities in Sediments of Two Disconnected Marginal Seas

Haixia Zhou, Hongyue Dang*, Martin G. Klotz

*** Correspondence: Hongyue Dang**: DangHY@xmu.edu.cn

#
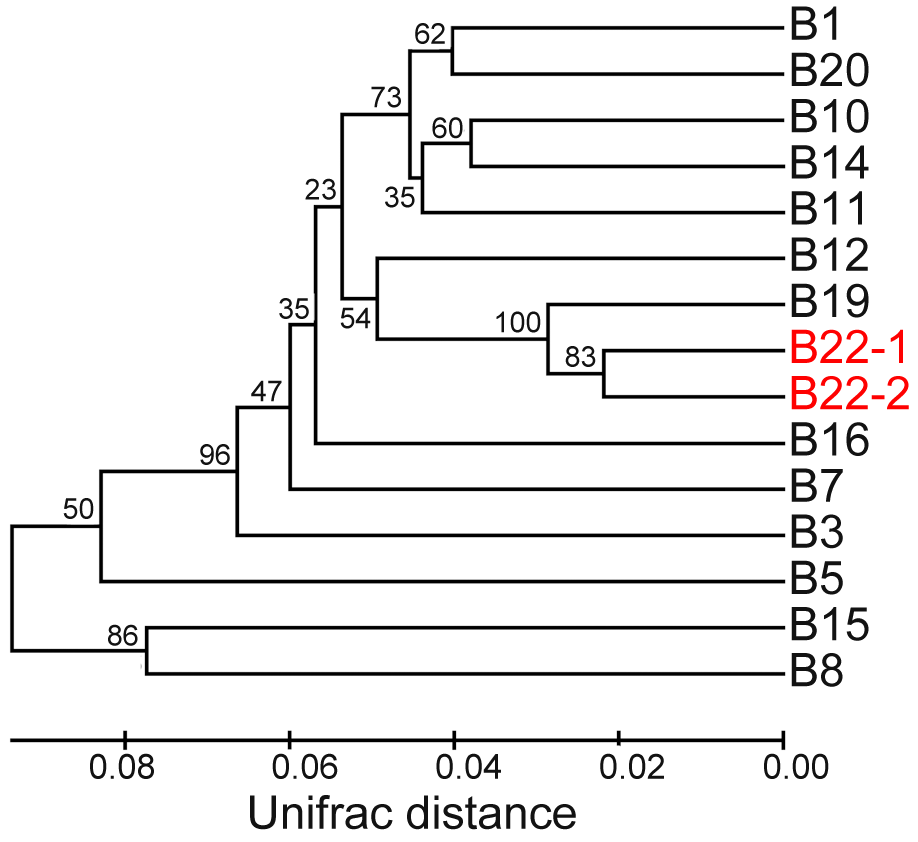


**Supplementary Figure S1 |** Hierarchical clustering dendrogram showing the similarity of duplicate *nifH*-harboring microbial assemblages in subcore samples B22-1 and B22-2 from Bohai Sea sediments. The dendrogram exhibiting the relationships between deduced NifH protein sequences was produced using the Fast UniFrac weighted and normalized Jackknife Environment Clusters method. The sequences representing *nifH* gene clone libraries from B22-1 and B22-2 sediment subcore samples at station B22 are clustered together tightly in the diagram, indicating that respective gene clone libraries of duplicate subcore sediment samples at the same station were highly similar.

**
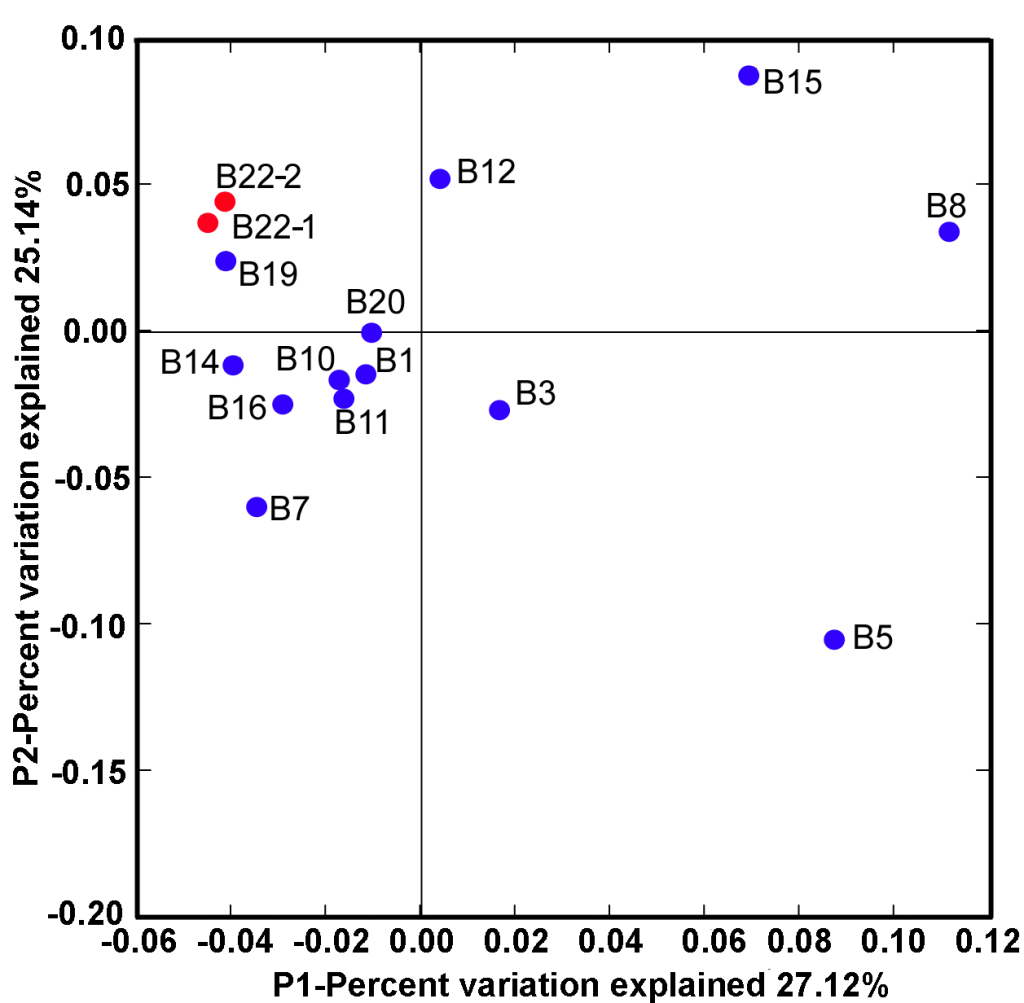
**

**Supplementary Figure S2 |** PCoA ordination diagram demonstrating the similarity of *nifH*-harboring microbial assemblages from subcore samples B22-1 and B22-2 from Bohai Sea sediments. This diagram was produced with the Fast UniFrac weighted and normalized PCoA method using the deduced NifH protein sequences. The two parallel *nifH* gene clone libraries of the B22-1 and B22-2 sediment subcore samples at station B22 are located very closely to each other in the diagram, indicating that the respective gene clone libraries of duplicate subcore sediment samples at the same station were highly similar.


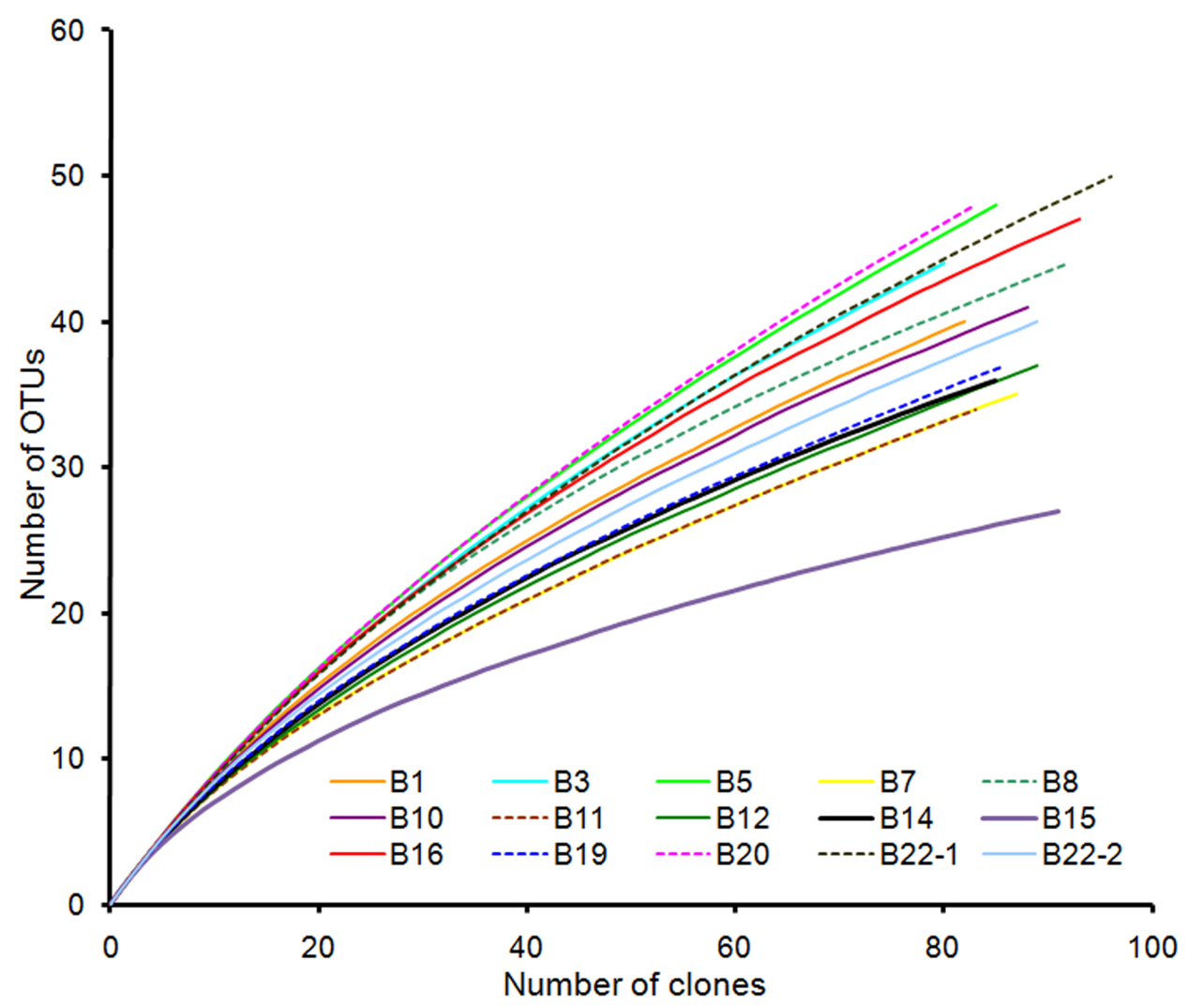


**Supplementary Figure S3 |** Rarefaction curves of the constructed *nifH* gene clone libraries using samples collected from Bohai Sea sediments.


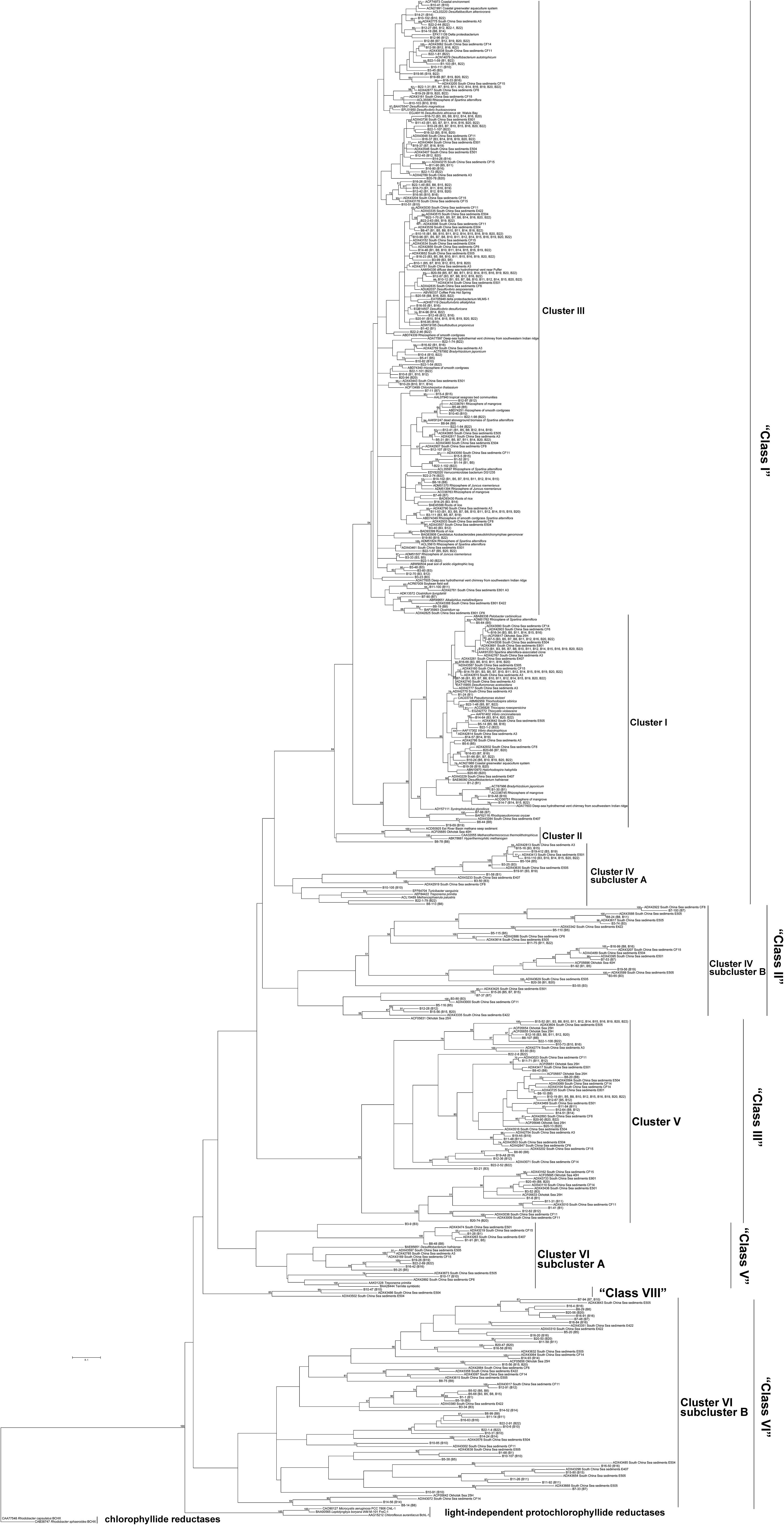


**Supplementary Figure S4 |** Phylogenetic tree of the Bohai Sea sediment NifH sequences (all the OTUs) constructed with the neighbor-joining method.


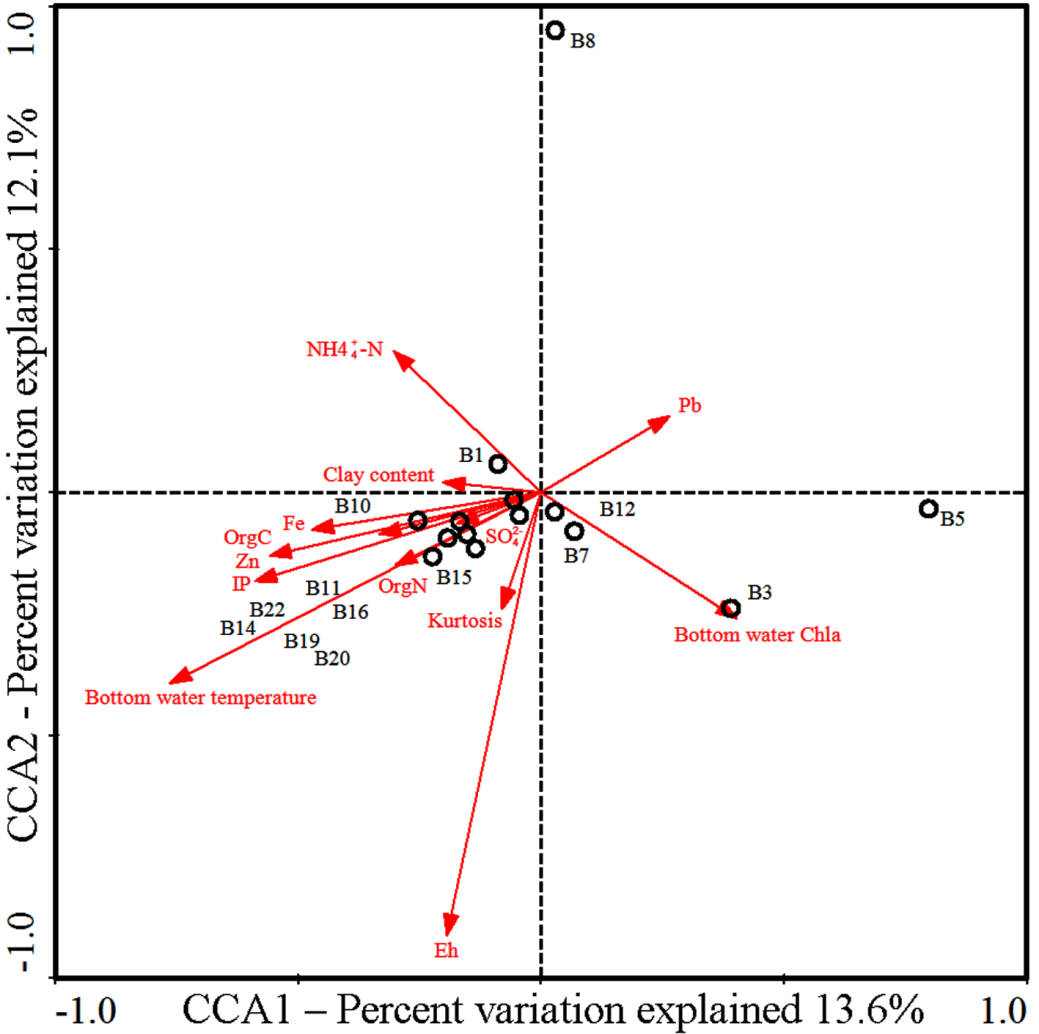


**Supplementary Figure S5 |** CCA ordination diagram of the relationship between the Bohai Sea sediment *nifH*-harboring assemblages and environmental factors. This diagram was obtained by using the NifH OTU data and only the first two principal dimensions of the CCA result were shown. Correlations between the Bohai Sea environmental factors and CCA axes are represented by the length and angle of arrows (environmental factor vectors) in the diagram. Covarying environmental variables (defined as r ≥ 0.950), such as bottom water chlorophyll a content and turbidity (r = 0.9551) and sediment porewater *Eh* and pH (r = -0.9999), were checked to minimize collinearity in the analysis.

**Supplementary Table S1 |** Measurements of *in situ* environmental parameters of the 14 sampling stations in the Bohai Sea.

| **Environmental**  **factor** | **Station** | | | | | | | | | | | | | |
| --- | --- | --- | --- | --- | --- | --- | --- | --- | --- | --- | --- | --- | --- | --- |
|  | B1 | B3 | B5 | B7 | B8 | B10 | B11 | B12 | B14 | B15 | B16 | B19 | B20 | B22 |
| **Longitude (°E)** | 121.50 | 121.03 | 120.70 | 120.01 | 120.65 | 118.43 | 119.48 | 120.28 | 118.92 | 119.17 | 120.00 | 119.65 | 119.90 | 119.64 |
| **Latitude (°N)** | 40.43 | 40.00 | 39.67 | 39.00 | 38.89 | 38.45 | 38.43 | 38.36 | 38.23 | 38.30 | 38.02 | 37.92 | 37.91 | 37.96 |
| **Water depth (m)** | 13.72 | 29.96 | 30.26 | 20.60 | 37.91 | 17.90 | 23.88 | 28.20 | 16.91 | 19.89 | 18.30 | 17.10 | 17.40 | 17.30 |
| **Surface water (at 1.0 m depth)**  Temperature (°C) | 24.89 | 24.37 | 24.72 | 24.82 | 22.73 | 25.43 | 25.30 | 23.71 | 25.83 | 24.87 | 23.85 | 25.21 | 24.39 | 24.76 |
| Salinity (PSU*^a^*) | 30.04 | 31.51 | 31.44 | 31.31 | 30.86 | 31.34 | 30.51 | 31.20 | 31.44 | 31.54 | 30.23 | 30.06 | 30.15 | 30.02 |
| EC25*^b^* (mS/cm) | 46.32 | 48.40 | 48.27 | 48.08 | 47.71 | 48.05 | 46.93 | 48.06 | 48.15 | 48.38 | 46.70 | 46.31 | 46.52 | 46.31 |
| Density (σ_t_) | 19.63 | 20.89 | 20.74 | 20.61 | 20.88 | 20.45 | 19.87 | 20.86 | 20.41 | 20.77 | 20.08 | 19.55 | 19.86 | 19.66 |
| Turbidity (FTU*^c^*) | 4.38 | 0.48 | 0.44 | 0.32 | 0.69 | 5.49 | 0.71 | 0.38 | 12.67 | 4.97 | 2.02 | 3.11 | 4.78 | 2.27 |
| Chl-*a* (ppb) | 2.47 | 1.65 | 1.08 | 0.94 | 2.24 | 2.32 | 1.96 | 1.82 | 2.46 | 1.91 | 1.56 | 2.44 | 1.55 | 2.09 |
| **Bottom water**  Temperature (°C) | 24.90 | 18.94 | 18.82 | 23.20 | 18.79 | 25.40 | 20.19 | 21.95 | 25.78 | 24.30 | 23.80 | 24.50 | 24.40 | 24.01 |
| Salinity (PSU) | 30.05 | 31.61 | 31.65 | 31.32 | 31.12 | 31.35 | 31.43 | 31.21 | 31.45 | 31.56 | 30.24 | 30.03 | 30.15 | 30.15 |
| EC25 (mS/cm) | 46.34 | 49.36 | 49.45 | 48.30 | 48.71 | 48.08 | 48.89 | 48.31 | 48.16 | 48.49 | 46.72 | 46.35 | 46.53 | 46.58 |
| Density (σ_t_) | 19.64 | 22.45 | 22.51 | 21.09 | 22.11 | 20.47 | 22.00 | 21.35 | 20.42 | 20.96 | 20.10 | 19.74 | 19.86 | 19.97 |
| Turbidity (FTU) | 5.60 | 6.40 | 75.52 | 9.84 | 5.37 | 31.42 | 12.63 | 3.20 | 20.33 | 23.47 | 7.37 | 11.17 | 10.05 | 6.99 |
| Chl-*a* (ppb) | 2.22 | 1.30 | 6.12 | 1.63 | 0.92 | 3.91 | 1.64 | 1.15 | 3.10 | 3.00 | 1.89 | 2.23 | 2.17 | 1.78 |
| **Sediment**  Water content (%) | 2.50 | 3.91 | 23.19 | 13.93 | 16.25 | 32.41 | 26.65 | 16.79 | 5.48 | 23.44 | 19.56 | 1.46 | 19.32 | 7.09 |
| Chl-*a* (%) | 0.35 | 0.28 | 0.77 | 0.91 | 2.66 | 0.12 | 0.34 | 0.69 | 0.29 | 0.36 | 0.51 | 0.69 | 1.17 | 0.51 |
| Pha-*a* (%) | 1.35 | 1.22 | 2.65 | 2.73 | 7.24 | 0.78 | 1.36 | 2.23 | 1.19 | 1.55 | 1.99 | 2.31 | 3.47 | 1.99 |
| sulfide (μg/g) | 19.50 | 9.04 | 12.20 | 5.40 | 48.20 | 7.52 | 132.0 | 6.88 | 29.4 | 14.8 | 43.7 | 103.0 | 11.3 | 11.3 |
| OM (organic matter, %) | 1.51 | 2.86 | 4.38 | 1.46 | 3.29 | 5.79 | 4.92 | 2.65 | 3.74 | 4.13 | 2.13 | 1.11 | 3.10 | 3.64 |
| OrgC (organic C, %) | 0.63 | 0.62 | 0.88 | 0.89 | 1.07 | 2.01 | 1.41 | 0.72 | 1.75 | 1.82 | 2.27 | 1.76 | 1.15 | 1.51 |
| OrgN (organic N, %) | 0.07 | 0.05 | 0.10 | 0.10 | 0.07 | 0.24 | 0.13 | 0.02 | 0.16 | 0.08 | 0.22 | 0.09 | 0.14 | 0.07 |
| OrgP (organic P, μmol/g) | 1.78 | 2.68 | 2.76 | 2.49 | 5.19 | 6.09 | 5.00 | 2.06 | 5.33 | 5.98 | 4.10 | 2.56 | 4.30 | 2.78 |
| IP (inorganic P, μmol/g) | 9.72 | 8.17 | 12.45 | 12.68 | 12.63 | 23.07 | 18.56 | 13.47 | 15.24 | 18.80 | 20.10 | 16.99 | 21.28 | 18.08 |
| TP (total P, μmol/g) | 11.50 | 10.85 | 15.21 | 15.17 | 17.82 | 29.16 | 23.56 | 15.53 | 20.57 | 24.78 | 24.20 | 19.55 | 25.58 | 20.86 |
| OrgC/OrgN | 9.00 | 12.40 | 8.80 | 8.90 | 15.29 | 8.38 | 10.85 | 36.00 | 10.94 | 22.75 | 10.32 | 19.56 | 8.21 | 21.57 |
| Petroleum hydrocarbon (μg/g) | 14.2 | 15.1 | 12.3 | 20.3 | 18.2 | 15.7 | 16.8 | 13.3 | 17.2 | 17.2 | 20.9 | 21.2 | 15.5 | 18.6 |
| Hg (ng/g) | 50.0 | 13.0 | 19.0 | 6.0 | 25.0 | 16.0 | 22.0 | 28.0 | 52.0 | 57.0 | 40.0 | 70.0 | 43.0 | 54.0 |
| Cd (μg/g) | 0.22 | 0.19 | 0.14 | 0.12 | 0.12 | 0.13 | 0.18 | 0.09 | 0.16 | 0.14 | 0.13 | 0.12 | 0.10 | 0.12 |
| Pb (μg/g) | 25.6 | 27.0 | 23.0 | 21.4 | 24.0 | 24.8 | 22.9 | 18.0 | 19.0 | 24.2 | 20.8 | 20.7 | 18.6 | 20.2 |
| Cr (μg/g) | 49.5 | 43.4 | 57.3 | 45.9 | 54.4 | 76.3 | 76.2 | 48.8 | 69.2 | 75.3 | 59.2 | 65.1 | 53.6 | 61.7 |
| Zn (μg/g) | 70.0 | 50.6 | 70.4 | 48.5 | 60.7 | 85.7 | 87.7 | 52.6 | 75.0 | 85.3 | 87.7 | 69.4 | 55.9 | 66.6 |
| Cu (μg/g) | 19.5 | 17.2 | 24.0 | 13.8 | 19.4 | 30.6 | 29.6 | 17.6 | 26.9 | 30.3 | 26.7 | 25.8 | 18.7 | 22.5 |
| Co (μg/g) | 9.21 | 5.96 | 9.19 | 6.00 | 7.80 | 12.90 | 12.80 | 7.59 | 11.50 | 14.00 | 8.98 | 11.30 | 8.80 | 9.36 |
| Ni (μg/g) | 23.2 | 19.1 | 26.6 | 18.9 | 22.9 | 34.3 | 35.0 | 20.6 | 30.5 | 35.4 | 22.5 | 28.2 | 22.1 | 26.2 |
| As (μg/g) | 5.33 | 6.13 | 5.18 | 5.05 | 5.98 | 12.40 | 8.43 | 5.58 | 13.80 | 14.70 | 11.50 | 13.60 | 13.90 | 13.30 |
| Fe (mg/g) | 21.7 | 20.0 | 26.1 | 20.4 | 25.8 | 32.7 | 36.6 | 23.8 | 31.7 | 35.6 | 27.0 | 30.5 | 26.5 | 28.6 |
| Mn (μg/g) | 399.0 | 382.0 | 536.0 | 594.0 | 491.0 | 588.0 | 676.0 | 467.0 | 627.0 | 684.0 | 594.0 | 605.0 | 621.0 | 600.0 |
| V (μg/g)*^d^* | 64.0 | 58.0 | 70.0 | 55.0 | 66.0 | 84.0 | 85.0 | 60.0 | 79.0 | 60.0 | 85.0 | 73.0 | 87.0 | 74.0 |
| S (mg/g)*^d^* | 35.4 | 30.8 | 30.9 | 37.4 | 37.6 | 32.2 | 33.3 | 30.6 | 25.6 | 32.4 | 29.2 | 35.8 | 28.1 | 28.8 |
| Sand content (%) | 45.08 | 33.55 | 17.52 | 51.65 | 33.91 | 1.38 | 3.31 | 28.95 | 3.03 | 4.09 | 7.95 | 15.82 | 16.98 | 7.55 |
| Silt content (%) | 40.16 | 50.61 | 61.27 | 36.82 | 46.49 | 64.23 | 65.36 | 59.07 | 71.67 | 69.63 | 77.17 | 69.31 | 69.52 | 75.44 |
| Clay content (%) | 14.76 | 15.84 | 21.21 | 11.53 | 19.60 | 34.39 | 31.33 | 11.98 | 25.30 | 26.28 | 14.88 | 14.87 | 13.50 | 17.01 |
| Median grain size (ø) | 5.78 | 5.38 | 4.45 | 6.02 | 4.76 | 2.95 | 3.25 | 5.51 | 3.81 | 3.64 | 4.94 | 5.00 | 5.25 | 4.80 |
| Mean grain size (ø) | 5.00 | 5.26 | 5.91 | 4.60 | 5.47 | 6.91 | 6.73 | 5.11 | 6.33 | 6.40 | 5.65 | 5.58 | 5.38 | 5.78 |
| Sorting coefficient | 1.91 | 1.90 | 1.90 | 1.83 | 2.02 | 1.61 | 1.67 | 1.73 | 1.75 | 1.75 | 1.67 | 1.75 | 1.72 | 1.74 |
| Kurtosis | 2.58 | 2.32 | 1.82 | 3.39 | 1.91 | 2.12 | 2.04 | 2.94 | 1.88 | 1.91 | 2.50 | 2.32 | 2.68 | 2.18 |
| Skewness | 0.97 | 0.80 | 0.35 | 1.28 | 0.51 | -0.15 | -0.01 | 1.04 | 0.27 | 0.17 | 0.80 | 0.71 | 0.91 | 0.64 |
| **Sediment pore-water**  Salinity (‰) | 33.3 | 34.0 | 33.8 | 34.7 | 34.1 | 33.9 | 33.4 | 33.6 | 33.3 | 34.1 | 33.8 | 33.6 | 32.8 | 33.6 |
| pH | 7.71 | 7.65 | 7.87 | 7.95 | 8.91 | 7.68 | 7.72 | 7.65 | 7.41 | 7.65 | 7.74 | 7.84 | 7.66 | 7.49 |
| Conductivity (mS/cm) | 42.6 | 44.2 | 43.7 | 42.0 | 43.9 | 43.6 | 42.6 | 42.5 | 43.6 | 43.9 | 42.0 | 41.7 | 41.3 | 42.6 |
| *Eh* (mv) | -38.1 | -34.3 | -47.2 | -51.6 | -106.2 | -36.2 | -38.6 | -34.6 | -21.6 | -34.6 | -40.1 | -45.8 | -34.7 | -25.9 |
| DO (μM) | 0.21 | 0.15 | 0.12 | 0.17 | 0.12 | 0.18 | 0.10 | 0.17 | 0.20 | 0.09 | 0.17 | 0.16 | 0.18 | 0.15 |
| NO_3_^-^-N (μM) | 2.66 | 1.13 | 3.92 | 2.48 | 4.90 | 0.07 | 4.42 | 1.30 | 0.29 | 6.16 | 6.19 | 7.34 | 12.16 | 20.17 |
| NO_2_^-^-N (μM) | 1.18 | 0.86 | 0.64 | 1.12 | 0.93 | 1.08 | 0.82 | 0.94 | 0.86 | 0.91 | 1.08 | 1.27 | 1.46 | 1.55 |
| NO_x_^-^-N *^e^* (μM) | 3.84 | 1.99 | 4.56 | 3.6 | 5.83 | 1.15 | 5.24 | 2.24 | 1.15 | 7.07 | 7.27 | 8.61 | 13.62 | 21.72 |
| NH_4_^+^-N (μM) | 257.64 | 109.08 | 36.76 | 57.84 | 151.36 | 78.60 | 59.40 | 52.82 | 132.08 | 124.82 | 68.48 | 134.94 | 88.22 | 63.24 |
| DIN*^f^* (μM) | 261.48 | 111.07 | 41.32 | 61.44 | 157.19 | 79.75 | 64.64 | 55.06 | 133.23 | 131.89 | 75.75 | 143.55 | 101.84 | 84.96 |
| PO_4_^3-^ (μM) | 2.80 | 1.92 | 1.62 | 4.67 | 2.31 | 0.92 | 1.26 | 3.31 | 0.51 | 1.75 | 2.86 | 3.46 | 1.80 | 1.58 |
| N/P (DIN/PO_4_^3-^) | 93.39 | 57.85 | 25.51 | 13.16 | 68.05 | 86.68 | 51.30 | 16.63 | 261.24 | 75.37 | 26.49 | 41.49 | 56.58 | 53.77 |
| SiO_3_^2-^ (μM) | 113.43 | 94.44 | 87.02 | 131.65 | 153.77 | 71.04 | 79.75 | 137.47 | 101.61 | 114.19 | 99.05 | 189.21 | 105.46 | 130.08 |
| SO_4_^2-^ (mg/g) | 1.11 | 1.13 | 1.35 | 1.27 | 1.20 | 1.92 | 1.85 | 1.03 | 1.24 | 1.68 | 0.98 | 1.02 | 1.01 | 1.35 |

*^a^* PSU: Practical Salinity Unit;

*^b^* EC25: Electrical Conductivity calibrated at water temperature of 25 ºC;

*^c^* FTU: Formazin Turbidity Unit;

*^d^* These environmental factors were measured in the current study, and all the other measurements were obtained from a previous study (Dang et al., 2013);

*^e^* NO_x_^-^ was calculated as the sum of NO_2_^-^ and NO_3_^-^;

*^f^* DIN (the total dissolved inorganic N concentration) was calculated as the sum of NH_4_^+^, NO_2_^-^ and NO_3_^-^.

**Supplementary Table S2 |** The efficiency and sensitivity of individual qPCR standard curve determined via plasmid DNA.

| **Target gene** | **Primer set** | **Efficiency** | | |  | **Sensitivity (copy number/μl plasmid DNA)** | |
| --- | --- | --- | --- | --- | --- | --- | --- |
|  |  | R^2^ | Slope | Linearity range |  | Expected value | Obtained value |
| *nifH* | nifH-fw  nifH-rv | 0.993 | -3.237 | 10^1^-10^6^ |  | 2.162 × 10^6^ | 2.241 × 10^6^ |
| Bacterial 16S rRNA* | 341F  518R | 0.991 | -3.365 | 10^3^-10^9^ |  | 1.870 × 10^6^ | 1.837 × 10^6^ |

* The bacterial 16S rRNA gene qPCR data were obtained from a previous study (Dang et al., 2013).

**Reference**

Dang, H. Y., Zhou, H. X., Zhang, Z. N., Yu, Z. S., Hua, E., Liu, X. S., et al. (2013). Molecular detection of *Candidatus* Scalindua pacifica and environmental responses of sediment anammox bacterial community in the Bohai Sea, China. *PLoS ONE.* 8, e61330. doi: 10.1371/journal.pone.0061330

**Supplementary Table S3 |** The taxonomic affiliations of the most abundant Bohai Sea sediment OTUs (≥ 5 clones) to the NifH sequences of known bacteria.

| **Bacterial phylum or class** | **Bacterial species** | **GenBank accession #** | **Identity**  **%** | **# of**  **OTUs** | **# of**  **clones** | **# of OTUs (# of clones) at each sampling station** | | | | | | | | | | | | | |
| --- | --- | --- | --- | --- | --- | --- | --- | --- | --- | --- | --- | --- | --- | --- | --- | --- | --- | --- | --- |
|  |  |  |  |  |  | B1 | B3 | B5 | B7 | B8 | B10 | B11 | B12 | B14 | B15 | B16 | B19 | B20 | B22 |
| *Alphaproteobacteria* | *Bradyrhizobium japonicum* | ACT67986, ACT67992, ACT67991, ACT67971 | 90-100 | 9 | 59 | 3(6) | 2(9) | 2(7) | 2(5) | 1(5) | 3(4) | 1(4) |  | 2(4) | 3(5) | 2(2) | 3(4) | 1(1) | 2(3) |
|  | *Rhodopseudomonas oryzae* | BAF62116 | 95 | 1 | 1 |  |  |  | 1(1) |  |  |  |  |  |  |  |  |  |  |
| *Gammaproteobacteria* | *Thiocystis violascens* | EGZ42772 | 98 | 1 | 4 |  |  | 1(1) | 1(1) |  |  |  |  |  |  |  |  |  | 1(2) |
|  | *Vibrio cincinnatiensis* | AAF61402 | 95-98 | 3 | 18 |  | 1(1) | 1(2) |  | 1(1) |  |  |  | 1(2) |  | 1(2) |  | 1(5) | 2(5) |
|  | *Vibrio diazotrophicus* | AAF17302 | 92-94 | 4 | 8 | 2(2) |  |  | 2(2) |  |  |  |  |  |  | 1(1) | 1(1) | 1(1) | 1(1) |
|  | *Halorhodospira halophila* | ABM61068, ABN10970 | 94-95 | 3 | 8 |  |  | 2(2) |  |  | 1(2) |  |  |  |  |  | 1(1) | 2(2) | 1(1) |
|  | *Thiorhodospira sibirica* | ABM92959 | 94 | 1 | 3 |  |  |  |  |  |  |  |  | 1(2) |  |  | 1(1) |  |  |
|  | *Ectothiorhodospira haloalkaliphila* | ABN10975 | 90 | 1 | 2 |  |  |  | 1(1) |  |  |  |  |  |  |  |  | 1(1) |  |
| *Deltaproteobacteria* | *Desulfuromonas acetoxidans* | EAT15955 | 97-100 | 3 | 139 | 2(16) | 3(7) | 2(3) | 2(10) | 1(5) | 3(18) | 3(11) | 2(6) | 2(18) | 2(9) | 2(6) | 2(8) | 3(14) | 2(8) |
|  | *Desulfuromonas sp.* WTL | ALC17315 | 97 | 1 | 22 |  | 1(3) | 1(3) | 1(8) | 1(1) |  | 1(2) | 1(2) |  |  | 1(1) |  | 1(1) | 1(1) |
|  | *Desulfurivibrio alkaliphilus* | ADH87119 | 95-97 | 3 | 73 | 1(4) |  | 2(4) | 2(2) | 2(6) | 1(3) | 1(4) | 1(2) | 1(4) | 1(5) | 2(7) | 2(11) | 2(4) | 3(17) |
|  | *Desulfovibrio aespoeensis* | ADU62037 | 94-95 | 3 | 44 | 3(6) | 1(3) | 2(2) | 2(2) | 3(10) | 2(2) | 2(3) | 1(1) | 3(4) | 1(2) | 2(3) |  | 2(2) | 3(4) |
|  | *Desulfovibrio desulfuricans* | EGB14507 | 93-98 | 9 | 240 | 3(8) | 2(2) | 3(4) | 3(18) | 4(8) | 3(15) | 3(16) | 5(26) | 4(15) | 4(24) | 6(18) | 4(24) | 3(12) | 5(50) |
|  | *Desulfovibrio magneticus* | BAH75547 | 89-93 | 12 | 72 | 4(9) | 3(5) | 2(3) | 4(6) |  | 4(6) | 3(4) | 3(6) | 3(5) |  | 3(7) | 3(4) | 4(5) | 5(12) |
|  | *Desulfovibrio africanus* | EGJ49116 | 91 | 1 | 3 |  |  |  |  |  |  |  |  |  |  |  | 1(1) | 1(1) | 1(1) |
|  | *Desulfobacterium autotrophicum* | ACN14079 | 95 | 2 | 10 |  |  |  | 1(1) |  |  |  | 1(1) |  |  |  | 1(1) | 1(1) | 2(6) |
|  | *Desulfobulbus propionicus* | ADW19185 | 93-95 | 3 | 18 | 1(1) |  |  |  |  | 1(1) |  |  | 1(5) | 1(3) | 2(2) | 1(2) | 1(2) | 1(2) |
|  | *Desulfatibacillum alkenivorans* | ACL03220 | 90-93 | 7 | 16 | 2(2) |  |  |  | 1(1) | 2(2) |  | 1(1) | 1(1) |  |  |  |  | 4(9) |
|  | *Desulfobacter postgatei* | EHG07866 | 91 | 1 | 3 |  |  |  |  |  |  |  | 1(1) |  |  | 1(1) |  |  | 1(1) |
|  | *Pelobacter carbinolicus* | ABA89338 | 92-95 | 3 | 138 | 1(7) | 2(13) | 3(12) | 1(15) | 1(9) | 1(6) | 2(17) | 1(13) | 2(6) | 2(4) | 2(11) | 1(6) | 1(4) | 1(15) |
| *Chlorobi* | *Chlorobium phaeobacteroides* | ACE04669 | 91 | 1 | 1 |  |  |  |  |  |  |  |  |  |  |  |  |  | 1(1) |
|  | *Chloroherpeton thalassium* | ACF13499 | 88-93 | 4 | 20 |  | 1(1) |  | 1(1) |  | 2(5) | 1(1) | 1(2) | 1(1) | 1(1) | 1(4) |  | 2(2) | 2(2) |
| *Firmicutes* | *Desulfitobacterium hafniense* | BAE86060 | 94 | 1 | 1 | 1(1) |  |  |  |  |  |  |  |  |  |  |  |  |  |
|  | *Clostridium beijerinckii* | AAF77055 | 91 | 1 | 1 |  |  |  |  | 1(1) |  |  |  |  |  |  |  |  |  |
|  | *Clostridium kluyveri* | EDK33075 | 90 | 1 | 1 |  |  |  |  |  |  | 1(1) |  |  |  |  |  |  |  |
| *Verrucomicrobia* | *Verrucomicrobiae* bacterium | EDY82020 | 90-91 | 3 | 17 | 1(1) |  | 1(2) | 1(2) | 1(1) | 1(2) | 1(4) | 1(1) | 1(2) | 1(1) |  |  |  | 1(1) |
